# Femtosecond Laser Irradiation Induced Heterojunctions Between Graphene Oxide and Silver Nanowires

**DOI:** 10.3390/ma18143393

**Published:** 2025-07-19

**Authors:** Jiayun Feng, Zhiyuan Wang, Zhuohuan Wu, Shujun Wang, Yuxin Sun, Qi Meng, Jiayue Wen, Shang Wang, Yanhong Tian

**Affiliations:** 1 State Key Laboratory of Precision Welding & Joining of Materials and Structures, Harbin Institute of Technology, Harbin 150001, China; fengjy@hit.edu.cn (J.F.); wzy13313278190@163.com (Z.W.); hit_wzh@163.com (Z.W.); 24s009108@stu.hit.edu.cn (S.W.); sunyuxin_2004@163.com (Y.S.); 24b309085@stu.hit.edu.cn (Q.M.); tianyh@hit.edu.cn (Y.T.); 2 Zhengzhou Research Institute, Harbin Institute of Technology, Zhengzhou 450041, China; wenjiayue_ep@hit.edu.cn

**Keywords:** femtosecond laser, silver nanowire, graphene oxide, heterojunction

## Abstract

In this article, femtosecond laser scanning was used to create heterojunctions between silver nanowire (Ag NW) and graphene oxide (GO), resulting in a mechanical and electrical interconnection. Surface plasmon resonances (SPRs) were generated on the nanowire surface by using femtosecond laser irradiation, producing a periodically excited electric field along the Ag NWs. This electric field then interfered with the femtosecond laser field, creating strong localized heating effects, which melted the Ag NW and GO, leading to mechanical bonding between the two. The formation of these heterostructures was attributed to the transfer of plasmon energy from the Ag NW to the adjacent GO surface. Since the connection efficiency of the nanowires is closely related to the specific location and the polarization direction of the laser, FDTD simulations were conducted to model the electric field distribution on the surface of Ag NW and GO structures under different laser polarization directions, varying the lengths and diameters of the nanowires. Finally, the resistance changes of the printed Ag NW paths on the GO thin film after femtosecond laser irradiation were investigated. It was found that laser bonding could reduce the resistance of the Ag NW-GO heterostructures by two orders of magnitude, further confirming the formation of the junctions.

## 1. Introduction

With the rapid development of modern electronic components, the new generation of devices is advancing toward micro/nanoscale architectures, multifunctional integration, and multi-material systems. Carbon-based nanomaterials, particularly carbon nanofibers (CNFs), graphene, and GO, have attracted widespread interest among researchers in recent years owing to their extraordinary mechanical properties, thermal resilience, and good biocompatibility [1,2,3]. These materials are revolutionizing critical sectors in neuromorphic computing systems [4], green energy technologies [5,6,7], and implantable bioelectronics [8].

Nanoscale interconnection enables precise assembly of nanomaterials, offering unprecedented advantages in fabricating highly integrated devices such as field-effect transistors [9], pn-type rectifiers [10], and memristors [11]. The performances of interconnected nanomaterials are often lower than expected due to scale effects and intrinsic structural limitations, as well as poor heterogeneous bonding interfaces. Current heterogeneous nanoscale bonding techniques primarily involve two categories: interconnections of nanomaterials in flexible electronics (such as Ag, Cu) [12,13,14], and interconnections of nanostructures in micro/nanoscale fabrications [15,16,17]. To address these bonding challenges, researchers have developed multiple bonding methodologies, including nano-brazing [18], thermal fusion welding [19], electron beam welding [20], ion beam welding [21], and laser-assisted bonding [22,23]. For example, Chen et al. demonstrated low-resistance Ag nanowire networks through thermal annealing at 200–320 °C [24], while Lu et al. achieved cold welding of Au nanorods via tip-induced diffusion using scanning tunneling microscopy [25]. Alternative approaches include ultrasonic bonding of carbon nanotubes to Ti electrodes via Al_2_O_3_ probes [16] and vacuum high-temperature brazing of CNT bundles using Ag-Cu-Ti alloys [26]. Despite these achievements, each technique exhibits specific constraints: thermal annealing requires protective surface coatings, cold welding necessitates sub-10 nm materials, beam-based methods demand vacuum environments, and resistive heating depends on material conductivity. The practical application of these techniques is limited by the complexity of the physical/chemical processes involved and the need to carry out these operations in a highly controlled atmosphere. In contrast, laser-assisted bonding emerges as a superior solution with distinct advantages: high energy density, rapid heating kinetics, minimized thermal impact zones, and ambient atmosphere operation. Ghosh et al. successfully fabricated Ag-ZnO NW and ZnO NW-Au nanojunctions through precision laser spot welding [19]. Zou et al. achieved femtosecond laser-based heterogeneous bonding of Ag NWs and TiO_2_ NWs, resulting in heterojunctions with high mechanical binding strength [27]. Feng et al. performed femtosecond laser bonding between Ag NWs and carbon nanofibers, reducing the contact resistance by six orders of magnitude [28]. However, femtosecond laser joining also requires precise control of parameters, including power, working height, and scanning speed, to ensure appropriate energy input at the junctions.

In recent years, there have been significant breakthroughs in nanodevices utilizing metallic nanowire interconnections [29,30], while research on bonding between metallic nanowires and non-metallic two-dimensional nanomaterials remains scarce. Therefore, this study investigates the femtosecond laser-induced interconnection between Ag NWs and GO. The bonding mechanism relies on laser-induced localized surface plasmon resonance (LSPR) effects in Ag NWs, generating periodic hotspots that transfer energy to the underlying GO layer, enabling localized melting and interfacial fusion. In addition, finite-difference time-domain (FDTD) simulations were conducted to elucidate the electromagnetic field distribution and energy transfer dynamics at the heterojunction. Post-irradiation electrical characterization further validated the functional performance of laser-bonded Ag NW-GO architectures, demonstrating enhanced interfacial conductivity.

## 2. Materials and Methods

The Ag NWs (with an average diameter of 50 nm and a length of 30~50 μm) were purchased from Zhejiang Kechuang Advanced Materials Technology Co., Ltd. (Hangzhou, China). The GO solution was purchased from Suzhou TANFENG graphene Tech Co., Ltd. (Suzhou, China). First, the GO solution (5 mg/mL) was diluted in deionized water, and the Ag NW solution was diluted in an ethanol solution. Then, 20 μL of GO solution was spin-coated onto a glass substrate, followed by 20 μL of Ag NW solution after the GO layer dried. After completely drying in air, the sample was irradiated with low-power-density femtosecond laser pulses (wavelength: 1064 nm; pulse width: 255 fs; repetition rate: 1 MHz; scanning speed: 2.5 mm/s). The laser beam was focused with a spot diameter of 11 μm on the focal plane and was vertically incident onto the center of the Si substrate, scanning within a 3 × 3 mm area. The laser power density was set between 60 and 70 mW. During the experiment, the sample stage was precisely controlled by a computer-driven XYZ three-axis translation stage. Scanning electron microscopy (SEM, Gemini560, Jena, Germany) and transmission electron microscopy (TEM, Tecnai G2 F30, Waltham, MA, USA) were used to characterize the Ag NW-GO heterostructures. Additionally, an inkjet printer was used to print Ag NWs for electrical performance testing, and electrical characterization was performed using a Keysight 34465A digital multimeter (Santa Rosa, CA, USA).

## 3. Results and Discussion

### 3.1. Characterization of Heterojunctions Between Ag NW-GO

**Figure 1:**
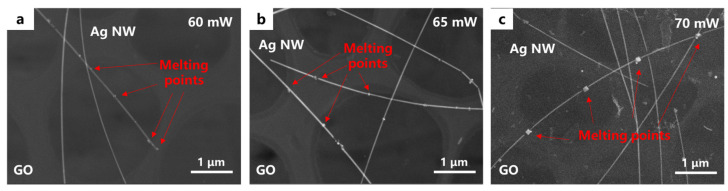
SEM images of the connection between graphene oxide and Ag NWs induced by the femtosecond laser. (**a**–**c**) Correspond to average powers of 70 mW, 65 mW, and 60 mW, respectively.

In this study, femtosecond laser-processed samples were observed using SEM. Figure 1 shows the SEM images of Ag NW-GO heterostructures formed at three different power settings. The femtosecond laser power parameters for Figure 1a–c are 70 mW, 65 mW, and 60 mW, respectively. It is evident from Figure 1 that some of the Ag NWs partially melted in the laser scanning area, while those in other regions remained intact. This phenomenon can be attributed to the LSPR effect of Ag NWs. Femtosecond laser excitation ionized surface electrons of Ag NWs, generating surface plasmons that interfered with the laser electric field, creating periodic electric field hotspots on the nanowire surface. These hotspots, through the heating effect of current, resulted in periodic melting points for the Ag NW. Additionally, the ends of the Ag NWs melted and merged into the GO, which indicates the softening and deformation of GO around the contact area, suggesting that the heterojunction formation principle under femtosecond laser irradiation involves the transfer of plasma energy from Ag NW to the GO surface. This enhanced the thermal effect at the Ag NW-GO interface, resulting in a larger interconnection area. Notably, a comparison of Figure 1a–c reveals that the higher the laser average power, the stronger the localized melting phenomena, with reduced GO surface ablation.

**Figure 2:**
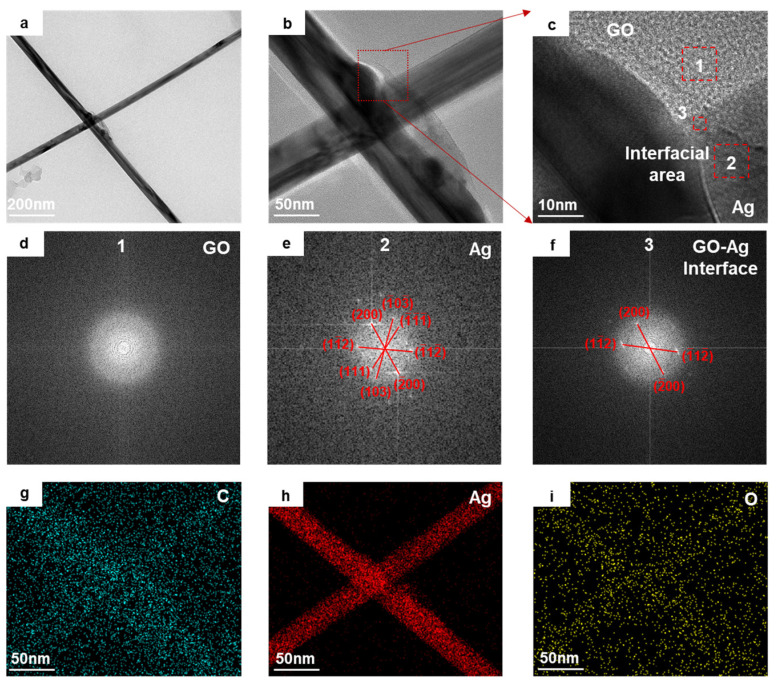
TEM images of the heterojunction at the intersection of Ag NWs and GO. (**a**,**b**) The overall morphology of the GO-Ag NW junction; (**c**) an HRTEM image at the junction; (**d**,**e**) the diffraction patterns of the GO and Ag NW in regions adjacent to the GO-Ag NW interface; (**f**) the diffraction pattern within the GO-Ag NW interface region; (**g**–**i**) EDX images of elemental abundance for C, Ag NW, and O in the junction region.

To demonstrate the heterojunction structures between Ag NW and GO, a detailed TEM analysis at the GO and Ag NW interface was conducted, where the bottom surface of the Ag NW contacted the top surface of the GO, as shown in Figure 2. From Figure 2a,b, it can be observed that the Ag NW melted and formed a constricted neck at the junction, while a small amount of Ag flowed aside, wetting and spreading to the GO surface. Additionally, a large area of amorphous material distributed along the Ag NW and covered the junction. A magnified high-resolution TEM (HRTEM) image of the interface between GO and Ag NW is shown in Figure 2c, and Fast Fourier Transform (FFT) analysis was performed on the regions of interest marked by dashed boxes 1, 2, and 3, which were confirmed to be GO, Ag NW, and their interface (GO-Ag), respectively, with the results presented in Figure 2d–f. Therefore, the amorphous material around Ag was proved to be carbon. The laser energy softened the GO around the Ag NW intersection area, causing the deformation of GO and the formation of a carbon layer on the Ag surface. The analysis revealed that GO exhibited an amorphous nature in regions of the heterostructure, whereas the Ag NW maintained its single-crystalline integrity. These findings also provide no evidence of element interdiffusion, intermetallic compounds, or lattice matching between GO and Ag NW at this interfacial area. So, it is assumed that GO and Ag NW were bonded by the amorphous carbon adhesive layer. According to the EDX spectra of elemental distributions (Figure 2g–i), it is evident that silver atoms did not diffuse into the GO matrix. Moreover, the laser irradiation may also have caused the surface oxidation of Ag, with the carbon (C) and oxygen (O) elements showing significant enrichment in the Ag NW region which are attributed to the remaining organic materials, silver oxide and amorphous carbon.

**Figure 3:**
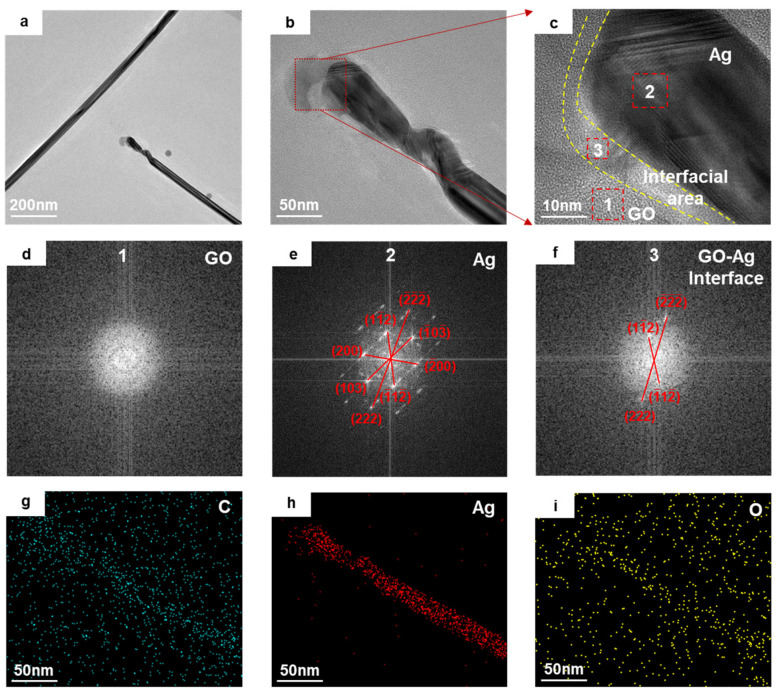
TEM images of the heterojunction at the endpoints of Ag NWs with graphene oxide (GO). (**a**,**b**) The overall morphology of the GO-Ag NW junction; (**c**) an HRTEM image at the junction; (**d**,**e**) the diffraction patterns of the GO and Ag NW in regions adjacent to the GO-Ag NW interface; (**f**) the diffraction pattern within the GO-Ag NW interface region; (**g**–**i**) EDX images of elemental abundance for C, Ag NW, and O in the junction region.

Figure 3 illustrates the heterojunction structures at the endpoints of the Ag NWs. The melting behavior observed here is similar to that at the intersections of Ag NWs shown in Figure 2. As seen in Figure 3b, melting occurred at the tip of a single nanowire. Figure 3c displays an HRTEM image of the interfacial region between the Ag NW and GO. The dashed box regions (GO, Ag, GO-Ag) in Figure 3c were subjected to FFT processing, and the results are shown in Figure 3d–f. These results demonstrate that atomic diffusion occurred at the interface without the formation of any new phases, thereby preserving the original lattice structures of both GO and Ag NW. This connection mode is consistent with the bonding observed in Figure 2b,c, which also indicates physical adhesive bonding, supported by the elemental distribution EDX spectra of the Ag NW and GO interface region (Figure 3g–i). Figure 3h,i demonstrate that C and O elements are enriched in the Ag NW region, while other areas maintain a uniform distribution, with no diffusion of Ag into the GO matrix (Figure 3i). This suggests that the connection at the endpoints of a single nanowire is similar to the melting regions of multiple intersecting nanowires. The changes caused by the LSPR effect are induced by the femtosecond laser, generating hotspots that melted localized regions of Ag NW and GO and leading to connections at the bottom of the Ag NW and the edges of the GO. The GO was softened and coated the edges of the Ag NWs.

### 3.2. Electric Field Simulation of Heterojunctions Between Ag NW and GO

By simulating the surface electric field distribution of GO and Ag NWs under femtosecond laser irradiation using the FDTD method, we can gain further insights into the electric field and energy distribution of these materials under optical excitation. The simulation included Ag NWs with different diameters (45 nm, 90 nm) and lengths (1 μm, 2 μm) positioned above the GO layer. The femtosecond laser used has a Gaussian beam profile, with a wavelength of 1064 nm and a pulse width of 255 fs. Previous studies have shown that when the polarization direction of the laser is parallel to the long axis of a single metallic nanowire, the local electric field strength and hotspot temperature on its surface are maximized. Furthermore, simulation results indicate that when the polarization direction of the incident laser is parallel to the long axis of the Ag NW, the electric field enhancement and heating effects are strongest at both ends of the nanowire, resulting in the highest temperature, while the effects are lower when the polarization is perpendicular. In studies of randomly oriented nanowires, the angle between the laser polarization direction and the long axis of the metallic nanowire influences the surface electric field distribution. By investigating this phenomenon, the simulations in this section set the laser polarization direction parallel and perpendicular to the long axis of the Ag NW. The electric field and hotspot distributions for other polarization directions will fall between these two cases.

**Figure 4:**
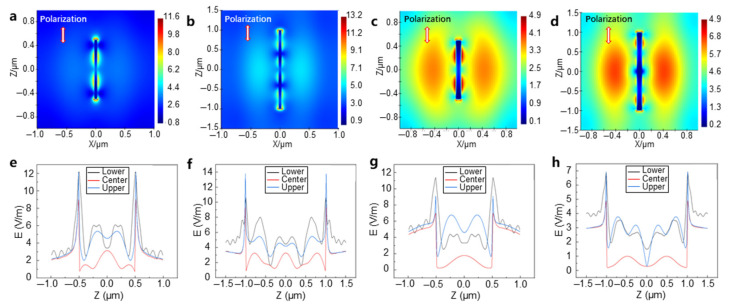
FDTD simulation of the electric field distribution under femtosecond laser irradiation of the GO-Ag NW heterostructure (with the femtosecond laser polarization direction parallel to the long axis of the nanowires). (**a**) A nanowire with d = 50 nm and L = 1 μm; (**b**) a nanowire with d = 50 nm and L = 2 μm; (**c**) a nanowire with d = 90 nm and L = 1 μm; (**d**) a nanowire with d = 90 nm and L = 2 μm. (**e**–**h**) The corresponding axial displacement–electric field strength distributions.

By varying the length and diameter of the Ag NW and simulating the electric field in the FDTD, with the laser polarization direction aligned with the nanowire’s long axis, the electric field distribution is shown in Figure 4. It is observed that under this laser polarization, the highest electric field enhancement occurs near the tips of the Ag NWs, which improves the heating efficiency of the heterojunction (Figure 4a–d). The field enhancement on the GO surface, in the absence of Ag NWs, is negligible, but significant enhancement is generated along the Ag NWs, creating periodic electric-field-strengthening regions. The field strength in the middle of the nanowire is lower than that at the ends (Figure 4a,b)). This indicates that most of the heat is generated by the excitation of the plasmon resonance effect in the Ag NWs. Comparing Figure 4a,c, the electric field intensity is more concentrated in the nanowire with a diameter of 50 nm. By comparing Figure 4a,b, we can observe that the 2 μm long nanowire exhibits more electric field concentration areas than the 1 μm nanowire. A detailed analysis of the electric field strength at the ends and middle regions of the nanowires shows that the highest electric field strength is observed at the ends. The electric field strength–axial displacement curves (Figure 4e–h) indicate that for the 1 μm nanowire, the electric field strength at the boundary in contact with the GO is higher than that at the center of the nanowire and the upper surface closer to the light source, suggesting that GO also affects the LSPR of silver (Figure 4e). Increasing the simulated nanowire length to 2 μm expands the difference in electric field strength between the ends and the middle region, and the areas of concentrated electric field strength increase (Figure 4f). When the diameter of the nanowire increases from 50 nm to 90 nm (Figure 4e,g), the LSPR effect weakens, the concentration of the electric field decreases, and the overall electric field strength reduces. It is hypothesized that the increase in diameter reduces the propagation efficiency of the plasmons, resulting in a weaker LSPR phenomenon.

**Figure 5:**
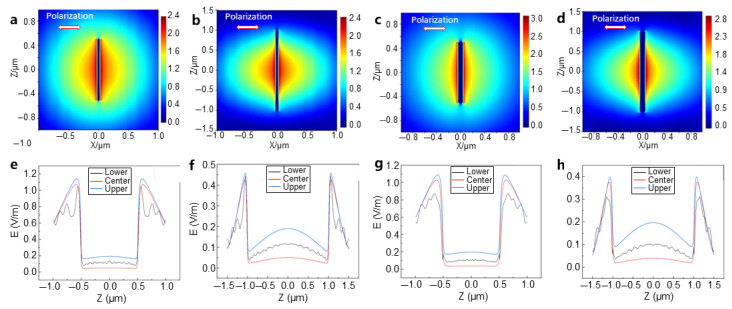
FDTD simulation of the electric field distribution under femtosecond laser irradiation of the GO-Ag NW heterostructure (with the femtosecond laser polarization direction perpendicular to the long axis of the nanowires). (**a**) A nanowire with d = 50 nm and L = 1 μm; (**b**) a nanowire with d = 50 nm and L = 2 μm; (**c**) a nanowire with d = 90 nm and L = 1 μm; (**d**) a nanowire with d = 90 nm and L = 2 μm. (**e**–**h**) The corresponding axial displacement–electric field strength distributions.

Figure 5 shows the electric field distribution when the laser polarization direction is changed to be perpendicular to the long axis of the nanowires. From Figure 5a–d, it can be observed that the thermal field distribution on the nanowire is broader and less concentrated compared to when the polarization is parallel to the long axis of the nanowire (Figure 4a–d). No periodic electric field concentration is observed, and the overall field strength is significantly lower, indicating a weaker plasmonic effect. This result is consistent with current research findings, and the field strength distribution aligns with the energy distribution of the Gaussian beam used in the simulation, as further shown in Figure 5e–h. After extending the length of the Ag NW (Figure 5e,f), the overall surface field strength decreases, but the distribution remains unchanged. When the diameter of the nanowire increases from 50 nm to 90 nm (Figure 5e,g), there are no significant changes in either the overall field strength or its distribution. Additionally, at the highest electric-field-strengthening regions at the ends of the nanowires, the field strength at the boundary in contact with the graphene oxide is lower than that in the center region of the nanowire, which is in stark contrast with the case of parallel polarization.

The laser used in the experiment was circularly polarized, and the nanowires were randomly oriented, so the observed phenomena should fall between the results of the two polarization direction simulations. From the two simulation results, it can be observed that the nanowires showing periodic electric field concentration have an angle that is either parallel or small with respect to the polarization of the light. The experimentally observed hotspot spacing of approximately 800 nm is in the same order of magnitude as the simulation result of about 500 nm for the polarization parallel to the long axis of the Ag NW, thus validating the simulation results. The simulation results correspond with the experimental findings, showing that the areas of melting in some nanowires initially occurred at the ends, and periodic melting happened along the Ag NW surfaces. Conversely, some Ag NWs showed no changes because their long axis was perpendicular to the polarization direction of the femtosecond laser, resulting in poorer heating effects.

In this study, the incident laser intensity is low, making single-photon absorption the dominant mechanism for converting photon energy into thermal heating. Within metallic nanostructures, single-photon absorption excites electrons in the conduction band. The excited electron gas then transfers its excess energy to the lattice, achieving thermal equilibrium in a femtosecond to picosecond timescale. Photo-absorption can also excite surface plasmons. When the plasma absorption frequency resonates with the photon frequency, the coupling of light into the nanostructure is significantly enhanced. The excitation of surface plasmons localizes and amplifies the electromagnetic field in these nanomaterials, contributing to localized heating and the subsequent joining of metallic nanostructures. In contrast, the non-metallic GO layer heats up as the absorbed laser energy eventually transfers to the lattice. Since GO exhibits no LSPR effect, heating results from the direct excitation of electrons, forming electron–hole pairs or excitons that deposit energy at localized lattice defect sites. By controlling spatial localization and the beam’s thermal profile, laser processing can soften and even melt the GO lattice while minimizing heating effects on the substrate. Joining occurs when adjacent nanostructures are heated to the melting temperature under laser irradiation and then merge or coalesce to form a bonded structure.

### 3.3. Electrical Properties of Ag NW-GO Heterostructures

**Figure 6:**
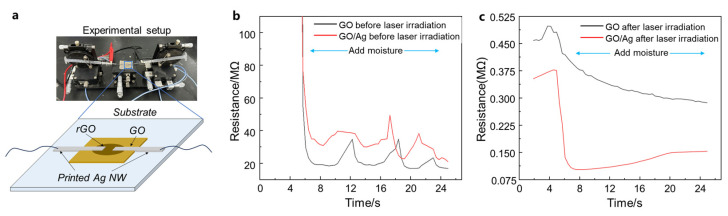
(**a**) An optical image and schematic diagram of the resistance measurement setup for the Ag NW-GO heterostructure; (**b**) the resistance response of GO and the GO-Ag NW heterostructure before femtosecond laser irradiation; (**c**) the resistance response of GO and the GO-Ag NW heterostructure after femtosecond laser irradiation.

The electrical properties of the Ag NW-GO heterostructures were measured using the structure shown in Figure 6a. The GO solution was drop-cast onto a glass substrate, and after drying, an Ag NW path was printed. In the joining experiment mentioned above, optimal laser parameters had already been determined, so the final laser power used was 60 mW. Figure 6a,b illustrate the resistance responses of GO and the GO-Ag NW heterostructures before and after femtosecond laser irradiation. Prior to laser irradiation and without moisture exposure, both pure GO and GO-Ag NW heterostructures exhibited infinite resistance, indicating an open-circuit state. The sheet resistance of the Ag NW network decreased as the overlap junction density increased, which ranged from 31 Ω∙sq^−1^ to 105 Ω∙sq^−1^. Moreover, the resistance of the structure was unstable and fluctuated with environmental vibrations, indicating poor mechanical stability. Upon introducing moisture at 5 s, a significant reduction in resistance was observed: the resistance of pure GO decreased to 2.0 ± 0.2 × 10^7^ Ω, while that of the GO-Ag NW heterostructure dropped to 3.5 ± 0.5 × 10^7^ Ω (Figure 6b). The higher resistance of the heterostructure compared to pure GO is attributed to the additional contact resistance between Ag and GO.

After laser irradiation and without moisture exposure, the resistance of pure GO was measured at 4.9 ± 0.3 × 10^5^ Ω and that of the GO-Ag NW heterostructure at 3.7 ± 0.2 × 10^5^ Ω, and the sheet resistance of the joined Ag NW network ranged from 2.5 Ω∙sq^−1^ to 10.3 Ω∙sq^−1^. The resistance of the structure was stable under mechanical vibration and insensitive to junction density. Upon moisture exposure, both resistances further decreased: the resistance of GO gradually dropped to 3.0 ± 0.1 × 10^5^ Ω, while that of the GO-Ag NW heterostructure decreased to 1.0 ± 0.3 × 10^5^ Ω (Figure 6c). Unlike the pre-irradiation scenario, the post-irradiation heterostructure exhibited lower resistance than pure GO. This is due to the localized thermal effects generated by the LSPR effect of Ag, which improved the contact between Ag and GO and further reduced GO to a graphene-like structure, thereby lowering the resistance. Comparing the pre- and post-irradiation results (Figure 6b,c), it is evident that femtosecond laser irradiation effectively reduced contact resistance. After moisture exposure, the resistance decreased by two orders of magnitude, demonstrating that femtosecond laser irradiation successfully established electrical connectivity between GO and Ag NW. For nanodevices made of GO-based functional nanomaterials, such as supercapacitors, self-powered devices, and chemical sensors, the improvement in interfacial conductivity can lower the interface barrier and help collect the electrical signals or energy within the GO functional layer, ultimately improving their performance.

## 4. Conclusions

In this paper, we achieved the interconnection between Ag NW and GO by using femtosecond laser scanning irradiation, resulting in heterojunctions with lower contact resistance. The interconnection can be controlled by adjusting the average power of the laser, and the optimal laser power for joining was confirmed to be 60 mW. The joining effectiveness of nanowires is closely related to their geometry, different regions, and the polarization direction of the laser. The formation of the heterostructure is due to the generation of surface plasmons on the nanowire, creating a periodically excited electric field on the surface, and the transfer of energy to the nearby GO surface. This field generated strong local heating effects, which melted the Ag NW, softened the GO, and formed a heterojunction through an adhesive amorphous carbon layer. Furthermore, FDTD simulations were performed to model the surface electric field distribution of the Ag NWs and GO structures with different lengths and diameters under different laser polarization directions, further confirming the experimental conclusions. This study also investigated the resistance changes in printed Ag NW pathways and GO structures before and after femtosecond laser irradiation. The results revealed that femtosecond laser irradiation reduced the resistance of the heterostructure without moisture exposure from infinity to 3.7 ± 0.2 × 10^5^ Ω, while the resistance of the moisture-exposed heterostructure decreased from 3.5 ± 0.5 × 10^7^ Ω to 1.0 ± 0.3 × 10^5^ Ω, representing a reduction of two orders of magnitude. These findings demonstrate that femtosecond laser joining can effectively improve the conductivity of Ag NW-GO heterojunctions.

## Data Availability

The original contributions presented in this study are included in the article. Further inquiries can be directed to the corresponding author.
